# A Tri-Band Cooled Receiver for Geodetic VLBI

**DOI:** 10.3390/s21082662

**Published:** 2021-04-10

**Authors:** José A. López-Pérez, Félix Tercero-Martínez, José M. Serna-Puente, Beatriz Vaquero-Jiménez, María Patino-Esteban, Pablo García-Carreño, Javier González-García, Óscar García-Pérez, Francisco J. Beltrán-Martínez, Carlos Albo-Castaño, Juan D. Gallego-Puyol, Isaac López-Fernández, Carmen Díez-González, Inmaculada Malo-Gómez, Laura Barbas-Calvo, Pablo de Vicente-Abad, José A. López-Fernández

**Affiliations:** Yebes Observatory, Centro de Desarrollos Tecnológicos, Instituto Geográfico Nacional, Ministerio de Transportes, Movilidad y Agenda Urbana, Cerro de la Palera s.n., Yebes, E-19141 Guadalajara, Spain; f.tercero@oan.es (F.T.-M.); jm.serna@oan.es (J.M.S.-P.); b.vaquero@oan.es (B.V.-J.); m.patino@oan.es (M.P.-E.); pablo.garcia@oan.es (P.G.-C.); j.gonzalez@oan.es (J.G.-G.); ogarcia@oan.es (Ó.G.-P.); francisco.beltran@oan.es (F.J.B.-M.); c.albo@oan.es (C.A.-C.); jd.gallego@oan.es (J.D.G.-P.); i.lopez@oan.es (I.L.-F.); c.diez@oan.es (C.D.-G.); i.malo@oan.es (I.M.-G.); l.barbas@oan.es (L.B.-C.); p.devicente@oan.es (P.d.V.-A.); jalfernandez@mitma.es (J.A.L.-F.)

**Keywords:** VLBI, geodesy, radioastronomy, radiotelescope, receiver, radiometer, feed, backend, correlation, VGOS, RFI

## Abstract

This paper shows a simultaneous tri-band (S: 2.2–2.7 GHz, X: 7.5–9 GHz and Ka: 28–33 GHz) low-noise cryogenic receiver for geodetic Very Long Baseline Interferometry (geo-VLBI) which has been developed at Yebes Observatory laboratories in Spain. A special feature is that the whole receiver front-end is fully coolable down to cryogenic temperatures to minimize receiver noise. It was installed in the first radio telescope of the *Red Atlántica de Estaciones Geodinámicas y Espaciales* (RAEGE) project, which is located in Yebes Observatory, in the frame of the VLBI Global Observing System (VGOS). After this, the receiver was borrowed by the Norwegian Mapping Autorithy (NMA) for the commissioning of two VGOS radiotelescopes in Svalbard (Norway). A second identical receiver was built for the Ishioka VGOS station of the Geospatial Information Authority (GSI) of Japan, and a third one for the second RAEGE VGOS station, located in Santa María (Açores Archipelago, Portugal). The average receiver noise temperatures are 21, 23, and 25 Kelvin and the measured antenna efficiencies are 70%, 75%, and 60% in S-band, X-band, and Ka-band, respectively.

## 1. Introduction

Geodesy is the science that studies the orientation in space, the rotation, the shape and the size of planet Earth, together with the gravity field and the surface of the ocean floor. In particular, Space Geodesy is a sub-division of Geodesy for the measurement of Earth orientation in space and rotation through the observation of sources outside our planet, like artificial satellites or quasars. The geodetic Very Long Baseline Interferometry (VLBI) is a space geodesy technique that uses a global network of radio telescopes equipped with sensitive receivers on Earth to detect weak signals from far-away quasars required to carry out those geodetic measurements. This network operates under the umbrella of the International VLBI Service for Geodesy and Astrometry (IVS) (https://ivscc.gsfc.nasa.gov, accessed on 29 March 2021) [1].

In order to improve the accuracy of geodetic VLBI measurements from cm-level, as provided by current S/X systems, to less that 1 mm, a new generation of radio telescopes and receivers have been promoted and encouraged by the IVS and its members: the VLBI Global Observing System (VGOS) project [2]. Fast-moving radio telescopes of mid-range size (12–15 m) with broad-band receivers (2–14 GHz) are the key elements, so a large number of quasars can be observed every 24 h with larger signal bandwidths that provide better accuracy in the geodetic measurements. The Spanish response to VGOS is the so-called *Red Atlántica de Estaciones Geodinámicas y Espaciales* (RAEGE) project, in collaboration with Portugal [3].

The Yebes Observatory of the Spanish *Instituto Geográfico Nacional* (IGN) is a Technological Development Center of the IVS, and it has developed three simultaneous tri-band (S/X/Ka) low-noise cryogenic receivers for geodetic VLBI observations with VGOS-type radiotelescopes, as the ring-focus antennas of the RAEGE network project.

The tri-band receivers reported in this paper were designed as a first-light receiver for RAEGE / VGOS radio telescopes, to be used for their accurate radiometric characterization during commissioning, and to be compatible with legacy S/X systems, so an accurate measurement of the radio telescope position and a corresponding time series can be obtained through join observations inside the IVS network, before becoming a VGOS station. In addition, simultaneous X/Ka operation is feasible for astrometric observations, too.

A special and particular feature of this receiver is that the whole front-end, including the feed, operate at cryogenic temperature in order to minimize the total receiver noise. In contrast, other receivers do not report such configuration [4].

The measured average receiver noise temperature for these receivers is 22.8 K for S-band, 24.5 K for X-band, and 28.6 K for Ka-band.

**Figure 1:**
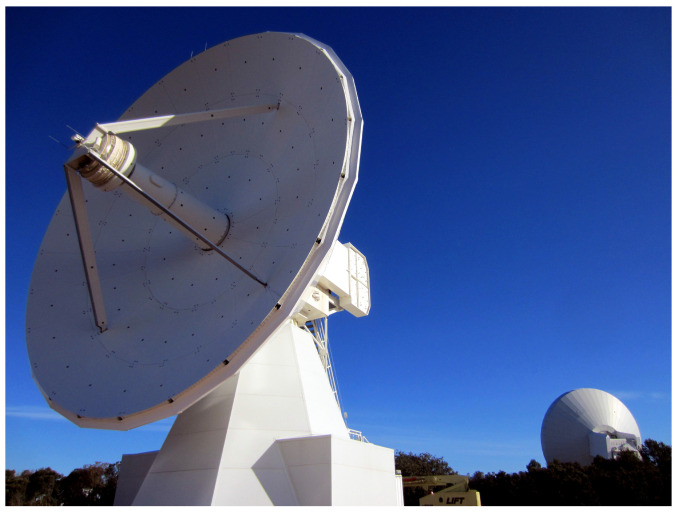
Yebes *Red Atlántica de Estaciones Geodinámicas y Espaciales* (RAEGE) VGOS (Very Long Baseline Interferometry (VLBI) Global Observing System (VGOS)) 13.2 meter radio telescope.

Three of these receivers were built in Yebes laboratories, as mentioned above. The first one was in operation in the Yebes 13.2 m RAEGE radio telescope (see Figure 1), until it was exchanged by a broad-band VGOS receiver (2–14 GHz). It is now borrowed by the Norwegian Mapping Authority (NMA) for the commissining of the Ny-Alesund radiotelescopes in Svalbard. The second one was developd for the 13.2 m VGOS antenna of the Geospatial Information Authority (GSI) in Japan. Finally, the third one is installed in the 13.2 m RAEGE antenna in Santa María (Açores archipelago, Portugal).

## 2. The Tri-Band Receiver

For the first light observations with the first RAEGE radio telescope, in Yebes Observatory, it was decided to develop a tri-band receiver [5], for which requirements were the following:

- Simultaneous S/X/Ka band reception;
- S-band range: 2.2–2.7 GHz;
- X-band range: 7.5–9 GHz;
- Ka-band range: 28–33 GHz;
- Simultaneous dual circular polarization in all bands;
- Mean receiver noise temperature <30 Kelvin;
- NoiseCal and PhaseCal injection;
- Cooled receiver feed;
- Cooling time <12 h.

The receiver consists of the following modules:

- The cryostat (dewar) with the cryo-cooled front-end (feed horn, hybrids, couplers, and low noise amplifiers);
- S-band downconverter;
- X-band downconverter;
- Ka-band downconverter;
- RF-over-fiber optical links for IF signal transportation through a 420-m-length fiber optic cable;
- NoiseCal and Phasecal Antenna Unit, for calibration signals generation, including 80 Hz switching of the noise source for continuous amplitude calibration;
- 5 MHz distributor for downconverters’ reference frequency;
- PhaseCal Ground Unit for the measurement of the delay introduced by the 5 MHz reference cable.

**Figure 2:**
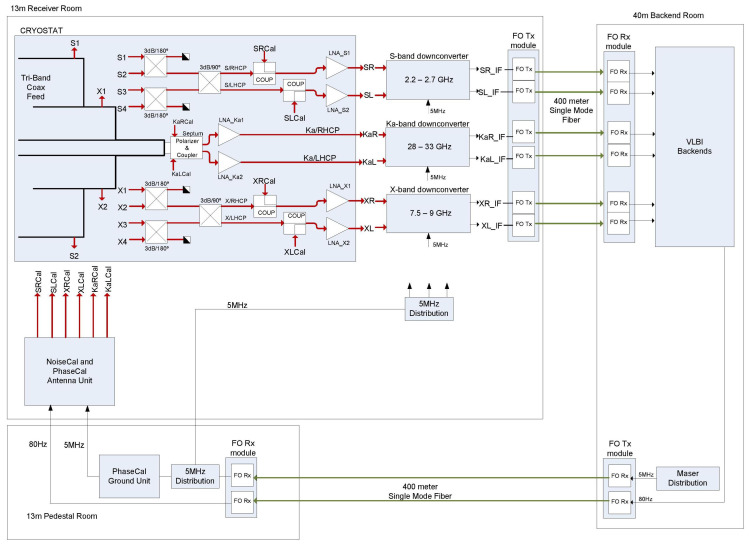
Tri-band receiver block diagram.

The full tri-band receiving system is depicted in Figure 2.

### 2.1. Receiver Feed

**Figure 3:**
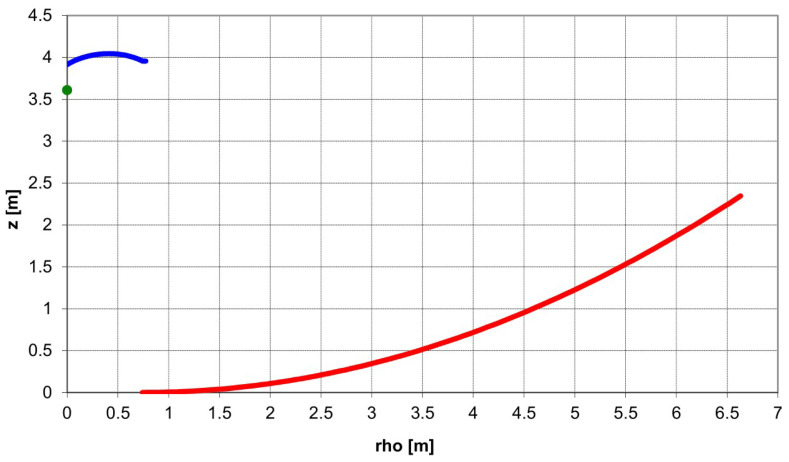
RAEGE radio telescope reflector profile.

The VGOS specifications require broad-band operation of the receiver from 2 to 14 GHz. There are several feeds to be used as a broad-band feed in VGOS project [6,7,8,9]. All these feeds have different types of output polarization (simultaneous linear and circular) and different types of output interfaces to the low noise amplifier (single-ended and differential). However, what is relevant from the antenna design view point is that all these feeds have a similar f/D range of operation (f/D = 0.3), broad beamwidth, and low directivity. This characteristic is important to define the dual reflector antenna, because it is unusual to have such a low f/D in dual reflector design, due to the fact that they show high blockage inefficiencies. To solve this issue, the ring-focus design avoids the blockage both from the feed and the subreflector when feeds with low f/D are used. Figure 3 shows the profiles of the RAEGE 13.2 meter radio telescope. Additionally, amplitude distribution in the aperture plane of the main reflector is flatter, due to the dual-reflector geometry. Consequently, the illumination efficiency improves at lower edge taper, which improves the spillover efficiency, too. Therefore, the feed to be designed must illuminate with a −16 dB taper for the 65° semiangle at the edge of the subreflector. Phase center of the feed must be coincident with the secondary focus of the antenna.

**Figure 4:**
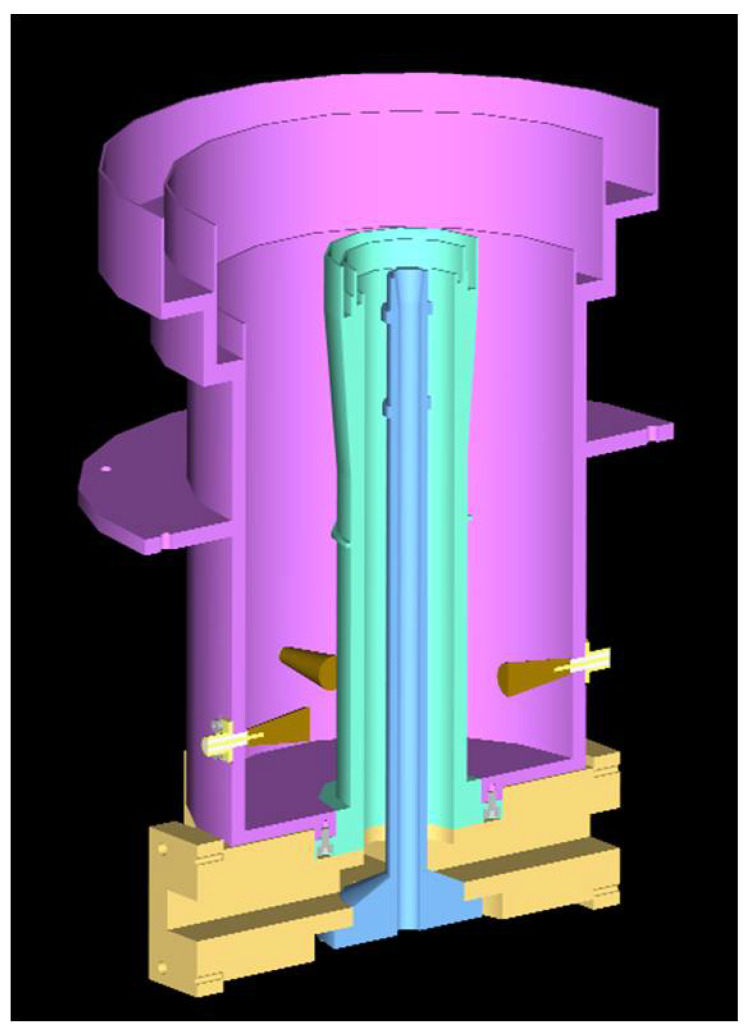
Internal view of tri-band receiver feed.

The tri-band receiver feed, which is shown in Figure 4, has been designed to illuminate the ring-focus antenna. It is made of a coaxial waveguide, for the S and X bands, and a circular waveguide for the Ka band. The biggest coaxial waveguide is the S-band one, whose inner conductor acts as the external one of the X-band waveguide. The same structure, with smaller dimensions, is repeated for the X band. In this case the inner coaxial conductor is the exterior one of the Ka-band. Finally, the smallest feed near to the axial axis is the Ka-band one, which is a single smooth conical feed. As the three feeds have the same beam-width, their apertures are scaled with the frequency. This fact makes possible the tri-band operation. The feed dimensions are 25 cm high and 20 cm in diameter, and it weighs 3 kg.

The three horns have independent outputs. In the case of the S-band coaxial horn, 4 coaxial field probes are combined to get dual-circular polarization. Opposite probes are combined in a 180° hybrid coupler to get one linear polarization. Both linear polarizations are then combined in a 90° hybrid coupler (see details in Section 2.3) to get dual circular polarization. For the X-band coaxial horn, similar layout is used but standard WR-112 to SMA-coaxial transitions are used. Then, 180° and 90° hybrid couplers are used the same way, to transform linear to circular polarizations. Since the 180° and 90° hybrids are connected with coaxial cables, they were phased matched to have the same electrical length, resulting in a low axial ratio in circular polarization. For the Ka-band feed, the output is a circular waveguide with 8.4 mm in diameter. It is directly attached to a septum polarizer which provides dual circular polarization in 2.92 mm coaxial connectors. This polarizer device includes a coupler for injection of noise and phase calibration signals.

**Figure 5:**
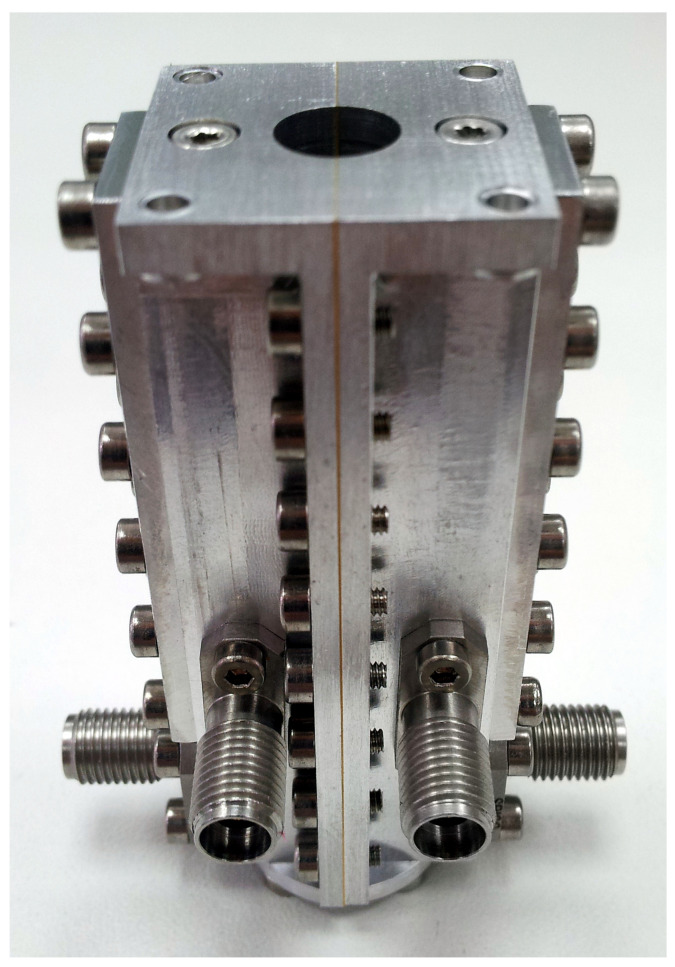
Ka-band septum polarizer and coupler.

The septum polarizer can be seen in Figure 5, where lateral coaxial connectors are for RCP and LCP outputs and front coaxial connectors are for calibration signal injection into the RCP and LCP chains. The circular waveguide port (top) connects to the Ka-band feed.

**Figure 6:**
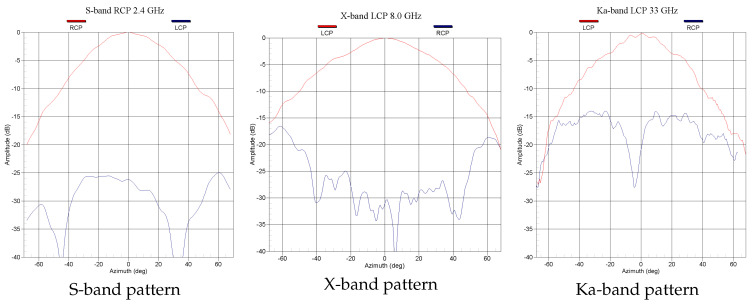
Measured radiation patterns of the tri-band feed in S, X, and Ka bands.

The tri-band feed radiation patterns were simulated using Ansys HFSS software. The feed was built and measured in the Yebes Observatory’s anechoic chamber. Figure 6 shows the measured radiation patterns at 2.4, 8, and 33 GHz, where a constant beam-width in the three bands to illuminate the ring reflector can be seen.

### 2.2. Receiver Cryostat

**Figure 7:**
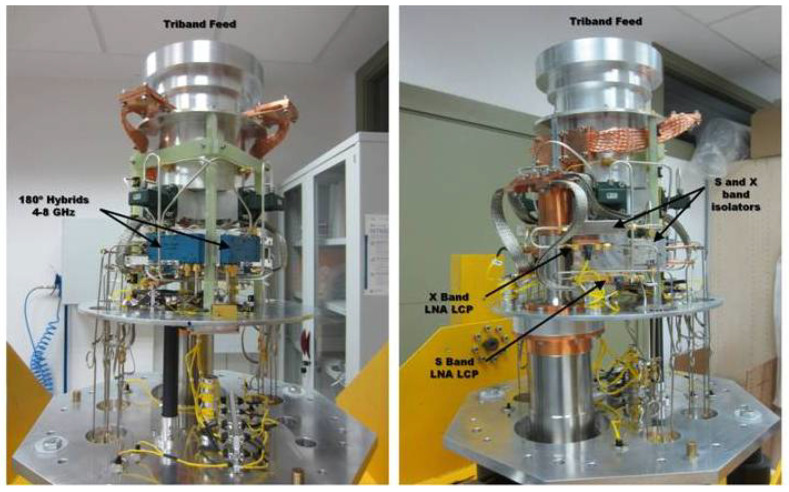
Receiver components inside de cryostat.

The cryostat is built over a two-stage closed-cycle refrigerator inside a stainless steel cylindrical dewar. Figure 7 shows the receiver components inside the cryostat from two different angles.

In order to characterize the vacuum and thermal behavior of the cryostat, a set of measurements was performed on the dewar. Table 1 summarizes the results of these measurements.

### 2.3. Hybrid Couplers

The receiver needs three cryogenic hybrid couplers for each band (S and X) to combine the signals from the feed probes (90° apart) to get the two ortogonal circular polarization signals, as requested in VLBI. Two of these couplers are 3 dB 180° hybrids, while the third one is 3 dB 90° hybrid.

The 3 dB 180° hybrids are commercial off-the-shelf units which have been tested a cryogenic temperatures with successful results. However, the 3 dB 90° hybrids have been designed in Yebes laboratories [10], and they are particularly designed for operation at 15 Kelvin.

**Figure 8:**
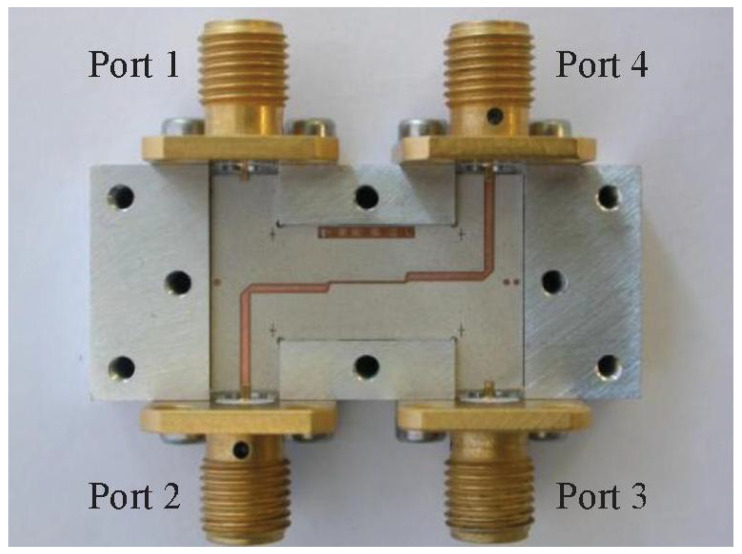
Partly assembled hybrid coupler.

The design of the 3 dB 90° hybrids couplers is based in stripline technology in order to achieve good couplings in a multioctave bandwidth, specifically, an offset broadside coupled stripline, with a dielectric separation layer (see Figure 8).

An example of the performance of a 3 dB 90° hybrid coupler for X-band is shown in Table 2, where the average equivalent insertion loss is given by $L_{eq}=10log_{10}\left(S_{11}^{2}+S_{12}^{2}+S_{13}^{2}+S_{14}^{2}\right)$.

### 2.4. Low-Noise Amplifiers

The cryogenic low noise amplifiers (LNAs) are key elements in defining the overall sensitivity of receivers, particularly in the current frequency range for which direct amplification of the sky signal is possible. Yebes Observatory has a long term experience in the design and manufacturing of state-of-the-art reliable cryogenic amplifiers.

**Figure 9:**
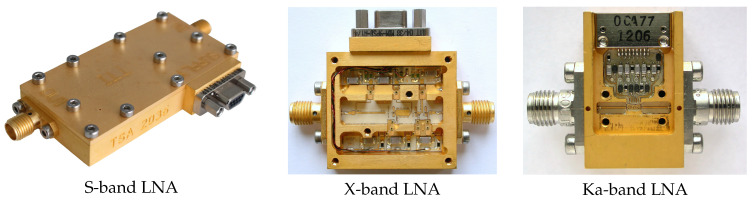
Cryogenic low noise amplifiers for the tri-band feed in S, X, and Ka bands.

The LNAs for the tri-band receiver are shown in Figure 9.

All amplifiers units were characterized at cryogenic temperature in the closed-cycle helium dewars of Yebes laboratories. Noise performance was measured using the cold attenuator method as described in Reference [11]. An absolute accuracy (2σ) at cryogenic temperature ($T_{amb}$ = 14 Kelvin) of 2.8 Kelvin for a typical Ka-band amplifier and of 1.4 Kelvin for a typical X-band amplifier can be estimated with methods presented in Reference [12]. Repeatability is better than these values by an order of magnitude.

On the one hand, S-band amplifiers were manufactured by Spanish company TTI Norte (www.ttinorte.com, accessed on 29 March 2021). The design is a legacy of the LNAs built for the low IF bands of HIFI (Heterodyne Instrument for the Far-Infrared, onboard the European Space Agency Herschel Observatory) [13].

On the other hand, X-band amplifiers benefited from the very successful design [14] made at Yebes Observatory for ALMA (Atacama Large Millimeter Array) band 9. The bandwidth required by the tri-band receiver (7.5–9 GHz) is narrower than the nominal band of those amplifiers (4–12 GHz). However, their performance is similar to narrowband designs in the VLBI/DSN X-band (8–9 GHz) [15]. The only drawback of this wideband approach is the need of an input isolator, as it is not optimized for low input reflection. The cryogenic isolators guarantee an optimal matching; however, this poses a penalty of about 1 Kelvin in noise temperature and increases a bit the complexity of the receiver.

Finally, Ka-band amplifiers are the outcome of a collaboration agreement between the IAF (Fraunhofer Institute for Applied Solid StatePhysics, Freiburg, Germany), the UC (Universidad de Cantabria, Spain) and Yebes Observatory. Within this framework, a 2.5 × 1 mm MMIC (Monolithic Microwave Integrated Circuit) was designed in the 25–34 GHz band with the aim of covering the needs of the radio astronomy and geodesy community, as well as the up and down links of the deep space communication antennas [16]. It is a design in coplanar waveguide technology with three stages of HFETs (Heterostructure Field Effect Transistors) profiting from the excellent metamorphic GaAs 100 nm process of IAF, competitive with InP (Indium Phosphide) analogs. Although the amplifier is used in the tri-band receiver with coaxial ports (2.92 mm connectors), it is prepared to exchange these connectors by coaxial to waveguide transitions. The noise temperature of the LNA improves 1 to 2 Kelvin in this configuration.

**Figure 10:**
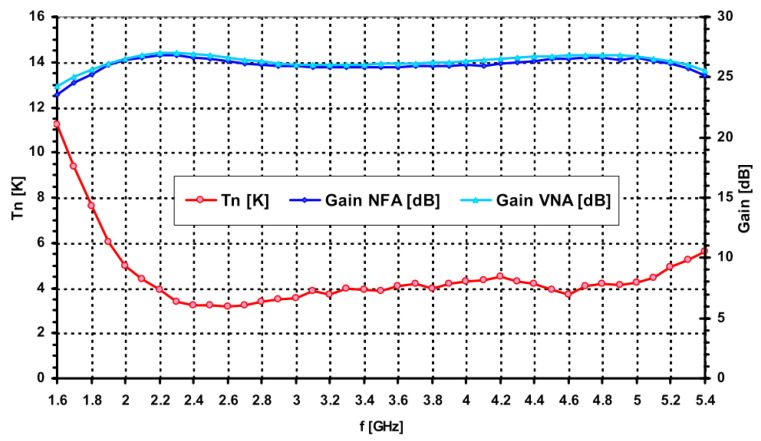
Gain and noise temperature plots of the S-band LNA at 14 Kelvin ambient temperature.

**Figure 11:**
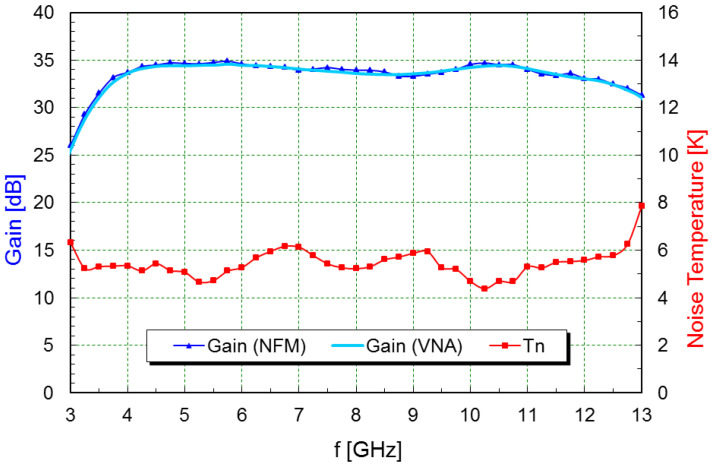
Gain and noise temperature plots of the X-band LNA at 14 Kelvin ambient temperature.

**Figure 12:**
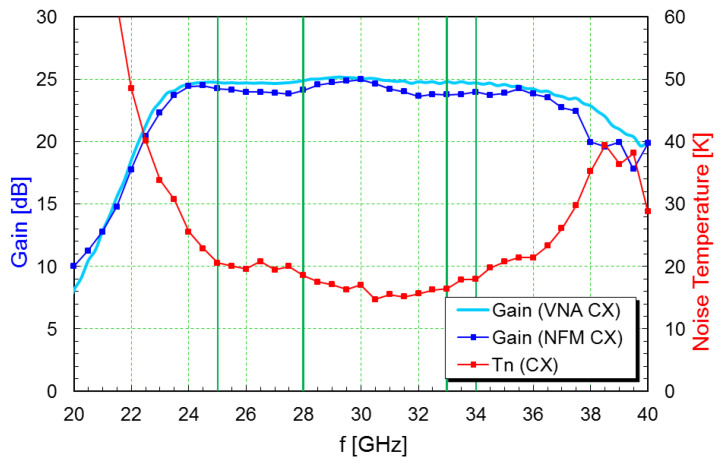
Gain and noise temperature plots of the Ka-band LNA at 14 Kelvin ambient temperature.

Plots with the noise and gain measurements at cryogenic temperature are shown in Figure 10, Figure 11 and Figure 12. A summary of the typical performance is presented in Table 3.

### 2.5. Frequency Downconverters

The output signals from the cryostat are sent to their corresponding room temperature downconverters for later amplification, filtering, and mixing. The final IF signal ranges from 500 to 1000 MHz, in S and Ka bands, and from 100 to 1000 MHz in X-band, as in classical geodetic VLBI receivers, to be backward compatible with older stations.

Table 4 shows a summary of the downconverter properties, as measured in the laboratory.

**Figure 13:**
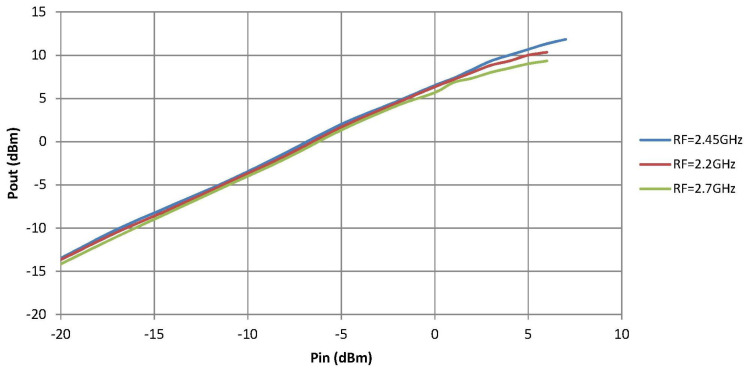
S-band downconverter linearity measurement.

Figure 13 shows the results of the linearity measurements of one of the S-band downconverter channels. The other channel is quite similar. From this figure, the Po1dB given in Table 4 is obtained.

### 2.6. Noise and Phase Calibration

It contains two noise sources, a common one for both S and X bands and another for Ka-band. These diodes can be remotely turned ON, OFF, or at 80 Hz rate. The signals from each source are combined with the train of pulses from the phasecal antenna unit and then splitted for injection in each receiver channel. The 5 MHz pulses in Ka-band are generated through up-conversion with an ultra stable 26 GHz DRPLO.

The phase calibration signal is generated with the help of ultra-fast logic gates fed with 5 MHz signal from the phase calibration ground unit, following a similar aproach to that shown in Reference [17].

## 3. Receiver Performance

**Figure 14:**
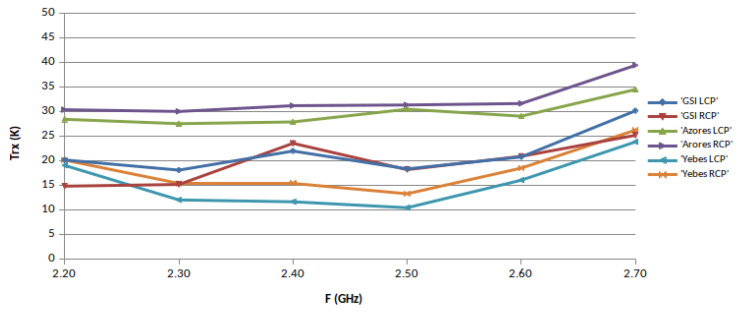
S-band receiver noise temperatures.

**Figure 15:**
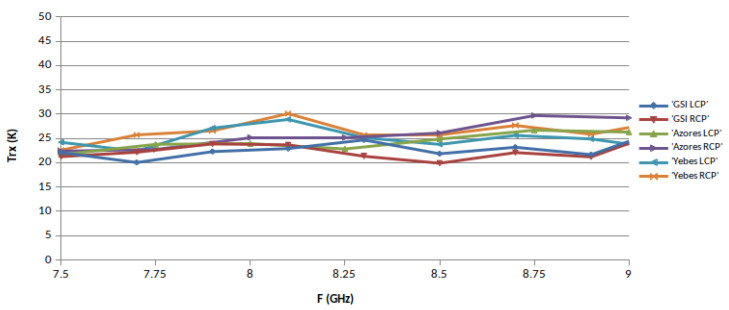
X-band receiver noise temperatures.

**Figure 16:**
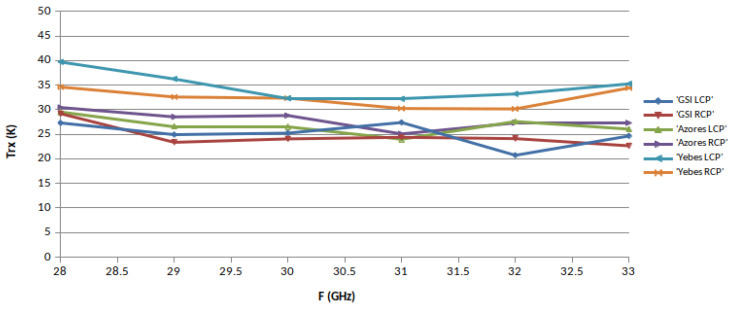
Ka-band receiver noise temperatures.

The noise temperature of the receivers, $T_{RX}$, was measured in each band in different ephocs using the Y-factor method, with microwave absorber submerged in liquid nitrogen (77.3 Kelvin) as cold load. The hot load was a piece of the same type of absorber at monitored room temperature. These loads were placed alternatively in front of the cryostat window to proceed with the measurements. The results are shown in Figure 14, Figure 15 and Figure 16, where the performance of the three receivers can be compared. It must be mentioned that in Figure 14, the noise increases at 2.4 and 2.7 GHz due to the existence of RFI.

The three receivers have a very similar performance, in particular in X-band. RAEGE Santa María receiver is worse in S and Ka bands for reasons that are not yet understood.

The average receiver calibration temperature, $T_{cal}$, was measured using the Y-factor method, too. It is the excess of noise added to the receiver by the noise diodes when they are ON (Section 2.6). It must be noted that the output power of the noise diodes was attenuated to get $T_{cal}$ values around 5% of $T_{RX}$.

The performance for Ishioka receiver is shown in Table 5.

## 4. Radio Telescope Performance with Tri-Band Receiver

The RAEGE Yebes radio telescope aperture efficiency, its system equivalent flux density (SEFD), the system noise ($T_{sys}$), and half-power beam width (HPBW) were measured by radiometric means with the tri-band receiver. Table 6 shows the results of these measurements.

Similar data for Ishioka and Santa María stations is not available, but theirs are expected to be quite similar, as all these radio telescopes have the same optical configuration. In fact, they were built by the same manufacturer.

## 5. Conclusions and Outlook

Yebes Observatory laboratories, in Spain, have developed a set of three simultaneous tri-band (S: 2.2–2.7 GHz, X: 7.5–9 GHz, and Ka: 28–33 GHz) low-noise cryogenic receivers for geodetic VLBI.

The first receiver was installed in the first radio telescope of the RAEGE network, which is located in Yebes Observatory, in the frame of VGOS project. After this, the receiver was borrowed by NMA for the commissioning of two VGOS radio telescopes in Ny-Alesund (Svalbard).

A second identical receiver was built for Ishioka VGOS station of GSI (Japan), and a third one for the second RAEGE VGOS station, located in Santa María (Açores archipelago, Portugal).

The average receiver noise temperatures of these receivers are 22.8, 24.5, and 28.6 Kelvin in S-band, X-band, and Ka-band, respectively, and the measured antenna efficiencies are 70%, 75%, and 60% in these three bands.

This type of receivers have proven to be very useful for the commissioning of VGOS radio telescopes, due to their high-frequency channel (28–33 GHz), and for initial VLBI tests, because they are backward compatible with legacy VLBI stations of the IVS network.

Currently, the receiver development activities at Yebes Observatory are focused in broad-band (2–14 GHz) receivers, and associated instrumentation, for VGOS project [18]. Additionally, a line of developments in high-temperature superconducting (HTS) filters for RFI mitigation has been started, too [19].

## Figures and Tables

**Table 1:** Cryostat performance.

Parameter	Value
Intermediate stage temperature	<33 Kelvin
Cold stage temperature	<16 Kelvin
Tri-band feed temperature	<9 Kelvin
Vacuum pressure	<$10^{-5}$ mbar
Leakage rate	<$1.8\times 10^{-5}$ mbar/s
Cooling time	<11 h
Warming time with heaters	<11 h
Warming time without heaters	<23 h

**Table 2:** X-band 3 dB 90° hybrid coupler performance.

Serial Number	YH90X	
Description	3 dB 90° cryogenic hybrid	
Frequency range	4–12 GHz	
Nominal coupling	3 dB	
Connector	SMA female, sliding pin	
Weight (typ.)	<36 g (1.27 oz)	
Temperature	297 Kelvin	20 Kelvin
Avg. equiv. insertion loss, $L_{eq}$	0.55 dB max	0.21 dB max
Max. return loss (any port)	−20 dB	−20 dB
Amplitude unbalance (max)	±0.27 dB	±0.3 dB
Phase unbalance (max)	±2°	±2°

**Table 3:** Typical performance of tri-band receiver low noise amplifiers (LNAs).

Frequency range (GHz)	2.2–2.7	7.5–9	28–33
Power dissipation (mW)	5.2	8.3	7.1
Average noise temperature (Kelvin)	3.4	5.5	16.4
Average gain (dB)	26.2	33.6	24.9
Gain flatness (dBpp)	0.7	0.4	0.5
Max. input return loss (dB)	−11	−3.3	−9.5
Max. output return loss (dB)	−16	−12.8	−12.3

**Table 4:** Summary of downconverter parameters.

Parameter	S-Band	X-Band	Ka-Band
Frequency range	2.2–2.7 GHz	7.5–9 GHz	28–33 GHz
First local oscillator	1.7 GHz	10–13 GHz	15–20 GHz
Second local oscillator	-	19.25 GHz	12.25 GHz
Output frequency range	500–1000 MHz	100–1000 MHz	500–1000 MHz
Gain	11.8–31 dB	12.2–31 dB	11.3–31 dB
Po1dB	+3 dBm	+1 dBm	0 dBm
Input matching	<−15 dB	<−20 dB	<−13 dB
Output matching	<−11 dB	<−21 dB	<−11 dB

**Table 5:** Average noise and calibration temperatures for Ishioka receiver.

	LHCP	RHCP	LHCP	RHCP
Frequency	*T_RX_*	*T_RX_*	*T_cal_*	*T_cal_*
Band	(Kelvin)	(Kelvin)	(Kelvin)	(Kelvin)
S (2.2–2.7 GHz)	21.5	19.6	0.8	0.9
X (7.5–9 GHz)	22.9	22.5	1.2	1.3
Ka (28–33 GHz)	25.0	24.6	1.6	1.6

**Table 6:** Radio telescope performance parameters.

Frequency	Efficiency	SEFD	*T_sys_*	HPBW
Band	(%)	(Jy)	(Kelvin)	(Arcmin)
S (2.2–2.7 GHz)	70	1700	50	42.5
X (7.5–9 GHz)	75	1300	40	11.3
Ka (28–33 GHz)	60	4000	100	3.2

## Data Availability

Data is available upon request to correspondence author.
